# Diagnosis and Treatment of Japanese Children with Neurogenic Bladder: Analysis of Data from a National Health Insurance Database

**DOI:** 10.3390/jcm12093191

**Published:** 2023-04-28

**Authors:** Takeya Kitta, Takahiko Mitsui, Naoko Izumi

**Affiliations:** 1 Department of Renal and Urologic Surgery, Asahikawa Medical University, Asahikawa City 078-8510, Japan; 2 Department of Urology, Graduate School of Medical Sciences, University of Yamanashi, Chuo City 409-3898, Japan; 3 Internal Medicine & Hospital Medical Affairs, Pfizer Japan, Inc., Tokyo 151–8589, Japan

**Keywords:** pediatric, neurogenic bladder, overactive bladder, insurance, administrative database, claims database, prescription, spina bifida, anticholinergic, Japan, urinary incontinence

## Abstract

In pediatric patients with neurogenic bladder (NGB), urinary tract evaluation, early diagnosis, and individualized management are important. We aimed to clarify the current status of diagnosis and treatment of NGB in Japanese children. This descriptive, observational, retrospective cohort study using the JMDC claims database included NGB patients aged ≤17 years over a 12-month follow-up period. Of the 1065 pediatric NGB patients, 38.9% had spina bifida. Dermatological and gastrointestinal comorbidities were common in the baseline period. Renal/bladder ultrasound was a commonly performed investigation (38.3%), but urodynamics was infrequently used (3.0%). Of all the overactive bladder medications, anticholinergics were used commonly (17.9% patients), and most patients used anticholinergics alone (without combination therapy). Clean intermittent catheterization (CIC; alone or in combination with medications) was performed in 9.3% of patients, and 3.9% of patients were concomitantly treated with medications. The most common incident complication was lower urinary tract infection (18.1%), which was especially common in patients with open spina bifida (54.1%). Despite guideline recommendations, lower urinary tract dysfunction is not thoroughly evaluated. Adequate understanding of patient status is critical to optimal patient management (behavioral therapy, CIC, and medication) in clinical practice.

## 1. Introduction

Neurogenic bladder (NGB) is a type of bladder and urethral dysfunction caused by a nervous system disorder affecting voiding and storage of urine [1]. Congenital diseases such as spina bifida and acquired ones such as spinal cord injury and central nervous system tumor are the most common causes of NGB in children [2]. Spina bifida, a congenital defect affecting vertebral fusion wherein vertebral arches forming the spinal canal are not fused properly, is further classified into open spina bifida (cystic spina bifida, represented by spinal meningiomas) and spina bifida occulta. Lower urinary tract dysfunction is almost inevitable (reported in >90%) in children with open spina bifida [3]. According to a Japanese statistical survey, the incidence of open spina bifida was 5.2 per 10,000 deliveries in 2012, although variations in the reports exist [4].

Children with NGB have reduced or no bladder sensation and present with urinary incontinence and detrusor overactivity. They are likely to develop bladder outlet obstruction, recurrent urinary tract infections, urosepsis, vesicoureteral reflux, and renal failure [5]. Bladder and urethral dysfunction lead to serious complications such as upper urinary tract diseases and severely affect the therapeutic outcome in children if appropriate urinary tract management is not provided. In the field of pediatric urology, at least 25% of diseases are due to NGB [6]. Pediatric NGB is diagnosed based on careful history taking, clinical examinations, and urodynamic investigations including urinalysis, ultrasound, and urodynamic assessment [7].

The goals of therapeutic management of NGB are to restore and maintain normal renal function, achieve or retain urinary continence, prolong the interval between catheterizations, prevent renal failure, reduce or eliminate vesicoureteral reflux and upper urinary tract dilatation, attain regular stool evacuation, and minimize the risk of urinary tract infection as well as improve the quality of life [8]. Early management of NGB in children involves a combination of non-pharmacological and pharmacological interventions. Clean intermittent catheterization (CIC) is the preferred method of bladder management for patients with NGB with partial or complete urinary retention. Anticholinergic medications are the first-line option for treating neurogenic detrusor overactivity [9]. When these fail, surgical interventions are considered.

In general, early diagnosis and treatment of NGB in children is challenging in clinical practice [10] due to the relative lack of specialists who are familiar with the protocols for management of pediatric NGB and the invasive nature of the tests. Recent Japanese guidelines [11] recommend early diagnosis of lower urinary tract dysfunction associated with spina bifida and suggest regular urinary tract evaluation and appropriate management with behavioral therapy, CIC, and anticholinergics, as needed. However, compliance with the guidelines in clinical practice may be limited by availability of fewer specialists, low awareness among healthcare professionals, and poor medical cooperation.

Thus, this study aimed to clarify the current status of clinical practice patterns for diagnosis and treatment of NGB in Japanese children by using the JMDC claims database (JMDC Inc., Tokyo, Japan) and consequently promote the importance of appropriate diagnosis and treatment. The specific objectives were to describe the following: demographics and baseline characteristics of children with NGB; implementation of clinical laboratory tests; disease management using medications and CIC; and incidence of complications.

## 2. Materials and Methods

### 2.1. Data Source

The data for this retrospective cohort study were extracted from the insurance claims database provided by JMDC Inc. The JMDC Claims Database is a national, anonymized claims database that has accumulated receipts (inpatient, outpatient, dispensing) and medical examination data received from a range of health insurance associations since 2005. This national, anonymized dataset provided monthly medical claims for Japanese company employees and their dependents aged 0–74 years, covered by employees’ insurance programs and included approximately 14 million (~11.1% of the total Japanese population) beneficiaries in February 2022 [12,13].

This dataset provided data on age; gender; eligibility gained/lost date; and inpatient and outpatient claims data including those of treatment (therapy, procedure), laboratory, and diagnostic tests performed (not the test results themselves), duration of hospitalization, medicines used (Anatomical Therapeutic Chemical Classification [ATC] code, product/brand, daily dosage for oral or intravenous medicines, duration), and diagnosis (disease codes according to International Classification of Diseases 10 [ICD-10]) by each item.

### 2.2. Study Design and Population

**Figure 1:**
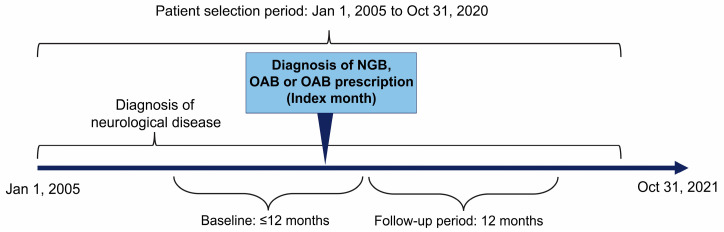
Study design. Baseline: The prior 12-month period including the index month for patients aged 1–17 years and the period from birth to the index month for patients aged <1 year. Follow-up period: The period of 12 months after the index month. Lost to follow-up was defined as patients leaving the JMDC database (no longer listed in the roster). NGB, neurogenic bladder; OAB, overactive bladder.

This was a descriptive, observational, retrospective cohort study (Figure 1). The target population was (1) children (aged ≤17 years) enrolled in the JMDC database with NGB diagnosis, overactive bladder (OAB) diagnosis, or prescription for OAB and (2) children (aged ≤17 years) with a diagnosis of neurological disease (as detailed below). The month of the first diagnosis of NGB, OAB, or prescription of OAB medicines was defined as the index month. Furthermore, patients had to meet all the following criteria to be eligible for inclusion in the study:

- One or more diagnosis of NGB or OAB (Table S1) or prescription of OAB medicines (Table S2) during the patient selection period (excluding suspected disease);
- One or more diagnosis of neurological disease (Table S3) that includes spina bifida, hydrocephalus, spinal cord injury, cerebral palsy, meningitis, brain tumor, or myelitis during the patient selection period (excluding suspected disease);
- Continuous registration in the JMDC database for ≥12 months before the index month including the index month for patients aged 1–17 years and from birth to the index month for patients aged <1 year; and
- Continuous registration in the JMDC database for ≥12 months after the index month.

No exclusion criteria were applied for this study.

### 2.3. Statistical Analysis

Pediatric NGB is a rare disease. Thus, the study was conducted without a formal sample size calculation and all eligible subjects were included. Categorical variables were reported using frequency and percentages, and continuous variables were reported using mean (standard deviation [SD]) or median and interquartile range (IQR), as appropriate. Where necessary, the date was imputed as the 15th in the month of medical treatment. Other missing values were not imputed.

Subgroup analyses were performed for predefined subgroup cohorts: Spina bifida cohort was defined as the cohort of all eligible patients with ≥1 diagnosis of spina bifida during the patient selection period (excluding suspected disease). This cohort was further subdivided into open spina bifida cohort (age <1 years at the first diagnosis of spina bifida) and spina bifida occulta cohort (age 1–17 years at the first diagnosis of spina bifida). Non-spina bifida cohort included all eligible patients with no diagnosis of spina bifida during the patient selection period. An unpaired *t*-test was used to compare continuous variables between cohorts and the chi-square test or Fisher’s exact test was used to compare categorical variables.

A multivariate analysis evaluating the potential risk factors for the development of urinary tract infection during the 12-month follow-up period (dependent variable) was performed. The diagnosis of spina bifida, patient demographics (sex and age at the index month), hospitalization and presence of comorbidities at the baseline, and use of CIC and/or OAB drugs at the index month were used as independent variables. A *p*-value less than 0.05 was considered to be statistically significant for all analyses.

All analyses were performed by the statisticians of JMDC. Analysis datasets were created using Amazon Redshift (ver.1.0.40677; Amazon Web Services, Inc., Seattle WA, USA), and statistical analyses were performed using SAS (ver.9.4; SAS Institute Inc., Cary, NC, USA) or JMP (ver.14.0; SAS Institute Inc., Cary, NC, USA).

### 2.4. Ethical Considerations

This study was conducted in accordance with the Ethical Guidelines for Medical and Health Research Involving Human Subjects issued by the Japanese regulatory authorities [14]. This study did not require institutional review board or independent ethics committee approval because the guidelines do not require such approval for information that has already been anonymized. This study did not require informed consent for the same reason.

## 3. Results

**Figure 2:**
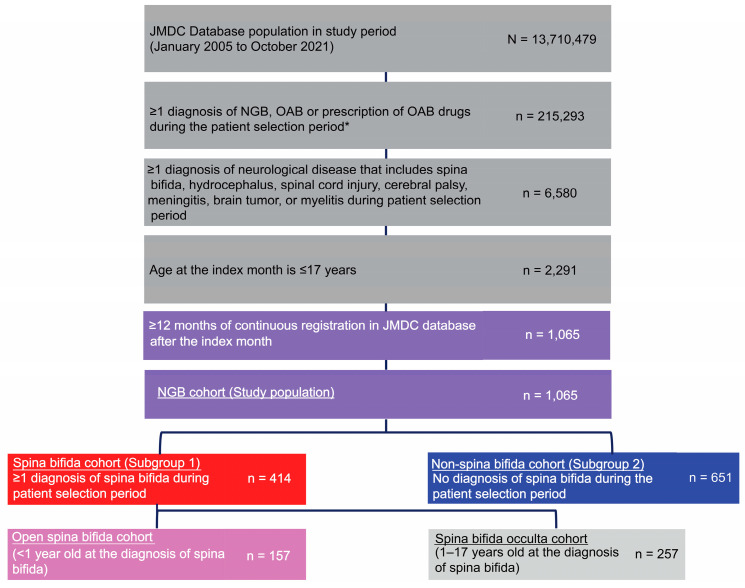
Patient flow. * The first month of diagnosis of NGB, OAB, or prescription of OAB drugs was defined as the index month. NGB, neurogenic bladder; OAB, overactive bladder.

Over the study period (1 January 2005, to 31 October 2021), 13,710,479 individuals were enrolled in JMDC (Figure 2).

Of these, 1065 individuals met the inclusion criteria: 414 children were in the spina bifida cohort and 651 in the non-spina bifida cohort. Within the spina bifida cohort, 157 children (37.9%) had open spina bifida and 257 children (62.1%) had spina bifida occulta. Most common diagnoses in the non-spina bifida subgroup were cerebral palsy (73.0%), brain tumor (7.7%), and meningitis (7.2%) (Table S4).

Table 1 presents the baseline characteristics of children with NGB according to the cohorts. The proportion of males was 59.4% in this cohort. The mean [SD] age of patients was 6.7 [4.8] years; the spina bifida cohort was significantly younger than the non-spina bifida cohort. Within the spina bifida cohort, the open spina bifida group was significantly younger than the spina bifida occulta group. The most common comorbidities diagnosed in the baseline were associated with the skin and gastrointestinal systems. These comorbidities were more common in the non-spina bifida cohort than the spina bifida cohort (see Table 1).

Specific laboratory tests to aid the diagnosis of NGB such as urodynamics, cystourethrography during urination, and static renal scintigraphy were not commonly performed in the index month and a month prior to that (Table 2). Renal/bladder ultrasound and urinalysis were performed in >30% patients, while magnetic resonance imaging (MRI) was conducted in 15.5% and urine culture and residual urine measurement in <10% patients. Generally, the proportion of patients undergoing most laboratory tests was higher in the spina bifida cohort than the non-spina bifida cohort. Within the spina bifida cohort, patients with open spina bifida were evaluated more commonly than those with spina bifida occulta. Notably, urinalysis, residual urine measurement, and urine flow measurement were performed more commonly in the spina bifida occulta than in the open spina bifida group.

Table 3 provides data on the treatment of NGB: 18.3% of patients received medicines (pharmacological treatment). The proportion of patients on medicines was significantly higher in the spina bifida vs the non-spina bifida cohort; the pattern persisted across types of medication, the number of medicines used, CIC, and concomitant use of CIC and medicines. Anticholinergic medicines (oxybutynin hydrochloride and propiverine hydrochloride) were the most commonly used. CIC was performed in 9.3% of all NGB patients, with about 40% of them being concomitantly treated with medicines. CIC was more commonly performed in the spina bifida (*p* < 0.01) cohort, specifically in the open spina bifida patients (*p* < 0.01).

Table 4 shows the incidence of the complications during the 12-month follow-up period. Lower urinary tract infection (18.1%), urinary incontinence (8.0%), and hydronephrosis (5.6%) were the most common incident diseases. Notably, 7.9% of patients had 4 or more repeated lower urinary tract infections in the cohort (data not shown). Subgroup analyses showed that the proportion of patients with 4 or more repeated lower urinary tract infections was significantly higher (19.1%) in the open spina bifida compared to the spina bifida occulta group (5.4%, *p* < 0.01) and the non-spina bifida cohort (6.1%, *p* < 0.01). The incidence of most complications was significantly higher in the spina bifida vs the non-spina bifida cohort. Furthermore, within the spina bifida cohort, the incidence of most complications was significantly higher in the open spina bifida than in the spina bifida occulta group (upper urinary tract infection and lower urinary tract infection). However, urinary incontinence was significantly higher in the spina bifida occulta than in the open spina bifida group.

**Figure 3:**
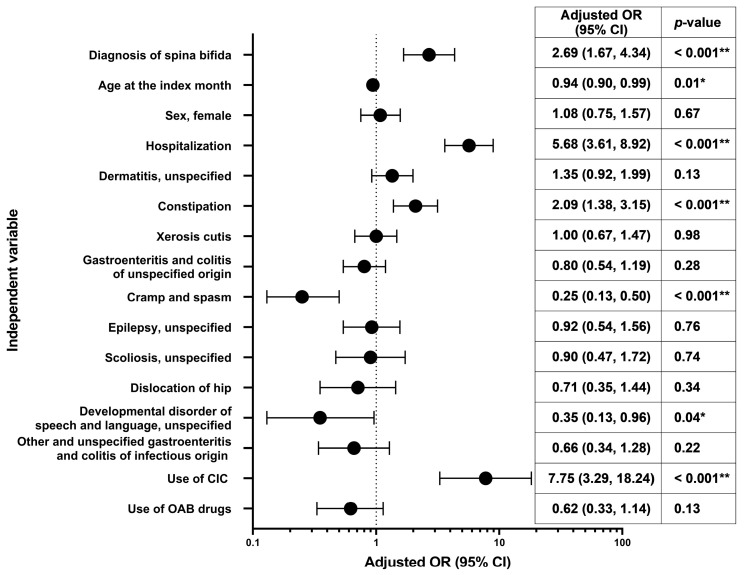
Multivariate odds ratios^†^ for development of urinary tract infection in the 12 months of follow-up. * *p* < 0.05, ** *p* < 0.001; ^†^ Adjusted for diagnosis of spina bifida (referent category: no), sex (referent category: male), age at the index month (continuous), hospitalization at the baseline, presence of comorbidities at the baseline (dermatitis, unspecified [L309]; constipation [K590]; xerosis cutis [L853]; gastroenteritis and colitis of unspecified origin [A099]; cramp and spasm [R252]; epilepsy, unspecified [G409]; scoliosis, unspecified [M419]; dislocation of hip [S730]; developmental disorder of speech and language, unspecified [F809]; other and unspecified gastroenteritis and colitis of infectious origin [A090]; referent category for each comorbidity: no), use of CIC at the index month (referent category: no), and use of OAB drugs at the index month (referent category: no). CI, confidence interval; CIC, clean intermittent catheterization; OAB, overactive bladder; OR, odds ratio.

Results of the multivariate analysis evaluating the risk factors for urinary tract infection during the 12-month follow-up period are shown in Figure 3. The multivariate odds ratios (ORs) were significantly higher with point estimates of ≥2 for CIC (7.75), hospitalization (5.68), presence of spina bifida (2.69), and constipation (2.09).

## 4. Discussion

This large-scale claims-based study revealed, for the first time, the characteristics of Japanese pediatric patients with NGB, the status of laboratory test implementation, and clinical practice patterns pertaining to treatment, as well as highlighted the potential concerns related to NGB management. This valuable evidence will pave the way for the development of future diagnosis and management practices for pediatric NGB.

### 4.1. Clinical Characteristics

Children with spina bifida comprised 38.9% of the NGB pediatric population, that is, it was the most common diagnosis in this group. The most common comorbidities diagnosed at baseline in our NGB cohort were associated with the skin (dermatitis, 43.1%; xerosis cutis, 36.7%), probably due to prolonged diaper use. In children with spina bifida, pressure ulcers are not uncommon as the spine and tailbone may protrude and rub against chairs and other surfaces. Pediatric NGB is known to be associated with neurogenic bowel dysfunction, leading to chronic constipation and stool incontinence [8]. Indeed, in our cohort, gastrointestinal comorbidities such as constipation (40.1%) and gastroenteritis and colitis (35.5%) were common. Therefore, patient/caregiver education for NGB should focus not only on urinary drainage but also on defecation care. Notably, 10.0% of the NGB cohort had disorders of language development. These were more common in the non-spina bifida cohort (14.6%), probably because the most common diagnosis in the non-spina bifida group was cerebral palsy, which in turn is associated with communication problems [15,16]. Thus, it is necessary to ensure clear communication with the affected children, family members, and educators to accurately identify and treat the patient based on urinary and other symptoms.

### 4.2. Diagnosis

Correct and timely diagnosis of pediatric NGB leads to appropriate management and optimal outcomes with minimal complications. The Japanese guidelines for the treatment of lower urinary tract dysfunction associated with spina bifida state that a detailed functional evaluation by urodynamics should be performed when neurogenic lower urinary tract dysfunction is suspected [11]. However, our study showed that though the renal/bladder ultrasound was performed in 61.6% of these patients, only 6.3% of patients with spina bifida underwent urodynamic evaluations. There could be several possible reasons for the low rate of urodynamics implementation, for example, difficulty in performing invasive urodynamics in children, low awareness among healthcare professionals, and inadequate medical coordination with specialists. Further investigation is needed to validate the causes of non-conformance with the guidelines. However, this finding is not surprising as guidelines are often not followed in real-world scenarios. Indeed, Kane et al. showed that European Society for Pediatric Urology (ESPU) members had large variations in clinical practice compared with recommendations by the European Association of Urology (EAU)/ESPU for the management of neurogenic bladder in children and adolescents [8,17]. In their online survey, Kane et al. included 103 ESPU members from 36 European countries [17]. During the initial inpatient stay of a newborn with spina bifida, all (100%) respondents carried out renal/ bladder ultrasound; however, 48% did a micturating/voiding cystourethrogram, 44% completed bladder volume measurements, while only 17% did videourodynamics. Their results should be evaluated in the light of the facts that most (80%) respondents of the survey were pediatric urologists and that the results of the survey may be affected by response bias as well as recall bias [17].

A UK general practitioners database-based study also revealed sub-optimal diagnosis of NGB among representative UK population [18]. The authors speculated inadequate medical coding, lack of awareness of urological symptoms among healthcare professionals, and failure to follow clinical guidelines in practice amongst the possible reasons for the sub-optimal diagnosis [18]. The same reasons may be applicable in the present pediatric population, and the numbers may be an underestimation of the real-world scenario.

### 4.3. Treatment

The Japanese guidelines highlight the importance of CIC and anticholinergics in the management of lower urinary tract dysfunction associated with spina bifida [11]. Self- or caregiver-administered CIC is a common treatment and the gold standard in the management of NGB patients with dysuria, a procedure that can be used safely and effectively across age groups for a long term. For functional lower urinary tract obstruction with a hyperbaric environment, CIC should be initiated immediately to avoid symptomatic urinary tract infections and renal dysfunction. Anticholinergic drugs should be administered as appropriate, usually in combination with CIC [11]. The recommendations from the EAU/ESPU guidelines are similar [8].

However, our results showed that these guidelines are not being followed in the real-world setting. Use of medicines was seen in 18.3% patients, more commonly in patients with spina bifida, especially in those with spina bifida occulta (35.0%). Spina bifida occulta differs from open spina bifida in that it rarely raises suspicion based on the appearance and is thus not diagnosed immediately after birth. Thus, the frequent use of medicines for urinary tract symptoms before diagnosis may result in more medicines used and fewer CICs performed. It is necessary to closely examine whether appropriate diagnosis is made and treatment is given. In the NGB cohort, among those who had received pharmacotherapy, anticholinergics alone were used in 95.9% of patients. CIC was performed in only 9.3% of NGB patients (most often in patients with open spina bifida [28.0%]). Concomitant use of CIC and medicines accounted for 3.9%. Kane et al., in their online survey study of ESPU members, reported that only 53% of the respondents had initiated CIC for all newborns with spina bifida during the initial inpatient stay [17]. However, 21% respondents started CIC after baseline urodynamics, and unfortunately, 19% initiated CIC only after abnormal changes were seen in renal/bladder ultrasound or videourodynamics. Similarly, 45% respondents did not follow the guideline recommendations for immediate initiation of anticholinergic medications on demonstration of bladder overactivity [17]. The high incidence of lower urinary tract infections indicates a need for better management of these patients. Individualized treatment using different categories of pharmacotherapies and surgical treatment may be needed [19].

### 4.4. Complications

In the 12-month follow-up period, urinary incontinence was present in 8.0% of the NGB patients and was more common in patients with spina bifida (15.9%). Furthermore, it was more common in the spina bifida occulta (24.9%) than in the open spina bifida (1.3%) group. In patients with open spina bifida, urinary incontinence may not be recorded accurately because patients are young and many may be regularly wearing diapers. Complications during the follow-up period included lower and upper urinary tract infections, urinary tract obstruction, vesicoureteral reflux, hydronephrosis, and renal failure, which were common in spina bifida patients, particularly in those with open spina bifida. The rate of lower urinary tract infection was high (18.1%) in all patients but was higher (29.2%) in the spina bifida cohort. Also, recurrent lower and upper urinary tract infections were more common in all patients.

Multivariate analysis evaluating risk factors for urinary tract infection showed significantly higher ORs with point estimates of ≥2 for CIC (7.75), hospitalization (5.68), presence of spina bifida (2.69), and constipation (2.09). This reaffirmed the findings of previous studies showing that those with CIC [20,21], spina bifida [21], and bowel and bladder dysfunction [22] were at risk of urinary tract infections. Additionally, hospitalization was also associated with urinary tract infection. However, given the limitation of the database wherein the cause of hospitalization remains unknown, it is difficult to establish the causal association and directionality. Also, some patients may have developed recurrent urinary tract infections, as described previously [20,23]. Our data on the complications of NGB suggest that management (monitoring and treatment) of NGB may be inadequate in routine clinical practice. Possible reasons could be lack of knowledge and awareness in physicians and co-medicals; fewer available specialists and/or lack of cooperation by them; and limitations of the healthcare system. Further investigation is needed to evaluate these causes and eliminate potential barriers in NGB management. The aforementioned complications not only reduce the quality of life of the patients and their families but also threaten life in severe cases. Therefore, early prevention and treatment of these complications are needed to improve patients’ quality of life. As recommended by the guidelines, it is necessary to perform laboratory assessments (such as urodynamics) to arrive at a timely diagnosis and to conduct regular follow-ups to monitor the patient and individualize the treatments.

### 4.5. Strengths and Limitations

Some strengths of our study should be highlighted. Large longitudinal database studies evaluating pediatric neurogenic bladder are scarce. Moreover, these available large studies are based largely on the adult population, with no discernible data for the pediatric age group [1,24]. This is the first Japanese large-scale real-world study on this clinically important condition in the pediatric population. Our study was performed using comprehensive data from the JMDC collected over a 15-year period. Specifically, the data can be tracked even if a patient transfers hospitals or uses multiple facilities. Thus, our results are likely to be generalizable to the Japanese pediatric population. Furthermore, our cohort of 1,065 children with NGB with 12 months of follow-up after the index month provides robust, retrospective, time-dependent data on various aspects such as diagnosis, management, and complications of NGB characterizing various subcohorts. Since the study was based on the administrative health database, potential recall bias was avoided.

The study also has some inherent limitations that should be acknowledged. Though we used comprehensive case definitions based on both diagnostic codes and prescription drug data, the possibility of misclassification cannot be ruled out in administrative databases [18]. Specifically, since medicines for OAB have been reported to be effective for NGB, it is expected that some NGB patients may have been designated as OAB patients due to the prescription of OAB medicines [18,25,26]. Also, there may be cases where patients who do not clinically have NGB are labelled as NGB for facilitating administration of medicines. Since the diagnosis name is based on the disease name described in the claims data, the possibility that it differs from the actual diagnosis cannot be denied. Furthermore, information prior to joining the healthcare insurance associations cannot be obtained. Therefore, it is possible that another neurological disease may be the cause of NGB. The laboratory test values are unavailable from the database and the purpose of conducting the tests remains unknown. Although the data on the prescription status of medicines are available, the actual medicine usage by the patients cannot be verified. Data on disease severity such as NGB are unavailable. Furthermore, in the present study, we did not assess if time trends for differences in the clinical practice patterns were evident over the follow-up duration. We plan to evaluate time trends, if any, in a subsequent analysis of these data.

## 5. Conclusions

Overall, our study shows that in routine clinical practice, lower urinary tract dysfunction is not often assessed per the recommendations from the guidelines. Without adequate evaluation, there is an increased risk of not appropriately identifying disease status, and therefore timely and appropriate treatment may not be provided. Thus, conducting periodic examinations, as recommended by the guidelines, and selecting the appropriate management strategy for each patient, including behavioral therapy, CIC, and medication, are necessary.

## Figures and Tables

**Table 1:** Patient demographics and baseline characteristics.

	NGB Cohort (n = 1065)	Spina Bifida			Non-Spina Bifida Cohort (n = 651)
		Total Cohort (n = 414)	(a) Open (n = 157)	(b) Occulta (n = 257)	
Age at the index month, mean [SD]	6.7 [4.8]	4.8 [4.5] **	0.7 [1.5] ^††^	7.4 [3.9]	7.8 [4.5]
Age at the index month, median (Q1–Q3)	6.0 (3.0–10.0)	4.5 (0.0–8.0)	0.0 (0.0–1.0)	7.0 (5.0–10.0)	7.0 (4.0–11.0)
Sex, male, n (%)	633 (59.4)	229 (55.3) *	75 (47.8) ^†^	154 (59.9)	404 (62.1)
**Comorbidities**, n (%)					
Dermatitis, unspecified	459 (43.1)	167 (40.3)	67 (42.7)	100 (38.9)	292 (44.9)
Constipation	427 (40.1)	107 (25.8) **	36 (22.9)	71 (27.6)	320 (49.2)
Xerosis cutis	391 (36.7)	133 (32.1) *	54 (34.4)	79 (30.7)	258 (39.6)
Gastroenteritis and colitis of unspecified origin	378 (35.5)	141 (34.1)	39 (24.8) ^††^	102 (39.7)	237 (36.4)
Cramp and spasm	257 (24.1)	2 (0.5) **	1 (0.6)	1 (0.4)	255 (39.2)
Epilepsy, unspecified	240 (22.5)	17 (4.1) **	6 (3.8)	11 (4.3)	223 (34.3)
Scoliosis, unspecified	188 (17.7)	11 (2.7) **	3 (1.9)	8 (3.1)	177 (27.2)
Dislocation of hip	165 (15.5)	6 (1.4) **	3 (1.9)	3 (1.2)	159 (24.4)
Developmental disorder of speech and language, unspecified	106 (10.0)	11 (2.7) **	2 (1.3)	9 (3.5)	95 (14.6)
Other and unspecified gastroenteritis and colitis of infectious origin	99 (9.3)	34 (8.2)	8 (5.1)	26 (10.1)	65 (10.0)
**Hospitalization**, n (%)	503 (47.2)	169 (40.8) **	119 (75.8) ^††^	50 (19.5)	334 (51.3)

The following ICD codes were used to identify specific comorbidities: L309, Dermatitis, unspecified; K590, Constipation; L853, Xerosis cutis; A099, Gastroenteritis and colitis of unspecified origin; R252, Cramp and spasm; G409, Epilepsy, unspecified; M419, Scoliosis, unspecified; S730, Dislocation of hip; F809, Developmental disorder of speech and language, unspecified; A090, Other and unspecified gastroenteritis and colitis of infectious origin. * *p* < 0.05, ** *p* < 0.01 between the spina bifida and non-spina bifida cohorts by the unpaired *t*-test, chi-square test, or Fisher’s exact test. ^†^ *p* < 0.05, ^††^ *p* < 0.01 between the open spina bifida and spina bifida occulta cohorts by the unpaired *t*-test, chi-square test, or Fisher’s exact test. ICD, International Classification of Diseases; NGB, neurogenic bladder; Q1, first quartile; Q3, third quartile; SD, standard deviation.

**Table 2:** Clinical laboratory tests conducted in the index month and 1 month prior to the index month.

	NGB Cohort (n = 1065)	Spina Bifida			Non-Spina Bifida Cohort (n = 651)
		Total Cohort (n = 414)	(a) Open (n = 157)	(b) Occulta (n = 257)	
**Specific tests**					
Urodynamics	32 (3.0)	26 (6.3) **	14 (8.9)	12 (4.7)	6 (0.9)
Cystourethrography during urination	12 (1.1)	4 (1.0)	4 (2.5)	0 (0.0)	8 (1.2)
Static renal scintigraphy	5 (0.5)	4 (1.0)	4 (2.5) ^†^	0 (0.0)	1 (0.2)
**Commonly performed tests**					
Urinalysis ^§^	354 (33.2)	181 (43.7) **	44 (28.0) ^††^	137 (53.3)	173 (26.6)
Urine culture	89 (8.4)	35 (8.5)	15 (9.6)	20 (7.8)	54 (8.3)
Renal/bladder ultrasound	408 (38.3)	255 (61.6) **	107 (68.2) ^†^	148 (57.6)	153 (23.5)
Residual urine measurement	83 (7.8)	47 (11.4) **	6 (3.8) ^††^	41 (16.0)	36 (5.5)
Urine flow measurement	57 (5.4)	41 (9.9) **	2 (1.3) ^††^	39 (15.2)	16 (2.5)
Magnetic resonance imaging	165 (15.5)	119 (28.7) **	67 (42.7) ^††^	52 (20.2)	46 (7.1)

Data are presented as n (%). ^§^ Since urinalysis is often encompassed at outpatient care in hospitals with more than 200 beds, the number of urinalysis cases may be less than the actual number of urinalysis cases. ** *p* < 0.01 between the spina bifida and non-spina bifida cohorts by the chi-square test or Fisher’s exact test. ^†^ *p* < 0.05, ^††^ *p* < 0.01 between the open spina bifida and spina bifida occulta cohorts by the chi-square test or Fisher’s exact test. NGB, neurogenic bladder.

**Table 3:** Treatment in the 12 months of follow-up: pharmacological treatment and CIC.

	NGB Cohort (n = 1065)	Spina Bifida			Non-Spina Bifida Cohort (n = 651)
		Total Cohort (n = 414)	(a) Open (n = 157)	(b) Occulta (n = 257)	
**Pharmacological prescription, n (%)**	195 (18.3)	123 (29.7) **	33 (21.0) ^††^	90 (35.0)	72 (11.1)
Anticholinergic drugs	191 (17.9)	121 (29.2) **	33 (21.0) ^††^	88 (34.2)	70 (10.8)
Oxybutynin hydrochloride	77 (7.2)	57 (13.8) **	28 (17.8)	29 (11.3)	20 (3.1)
Propiverine hydrochloride	67 (6.3)	45 (10.9) **	6 (3.8) ^††^	39 (15.2)	22 (3.4)
Solifenacin succinate	62 (5.8)	31 (7.5)	2 (1.3) ^††^	29 (11.3)	31 (4.8)
Imidafenacin	13 (1.2)	8 (1.9)	0 (0.0) ^†^	8 (3.1)	5 (0.8)
Fesoterodine fumarate	7 (0.7)	4 (1.0)	0 (0.0)	4 (1.6)	3 (0.5)
Tolterodine tartrate	2 (0.2)	2 (0.5)	0 (0.0)	2 (0.8)	0 (0.0)
Beta 3-agonists	5 (0.5)	3 (0.7)	0 (0.0)	3 (1.2)	2 (0.3)
Mirabegron	3 (0.3)	1 (0.2) **	0 (0.0)	1 (0.4)	2 (0.3)
Vibegron	2 (0.2)	2 (0.5)	0 (0.0)	2 (0.8)	0 (0.0)
Other (oral agents)	3 (0.3)	1 (0.2)	0 (0.0)	1 (0.4)	2 (0.3)
Flavoxate hydrochloride	3 (0.3)	1 (0.2)	0 (0.0)	1 (0.4)	2 (0.3)
**CIC, n (%)**	99 (9.3)	62 (15.0) **	44 (28.0) ^††^	18 (7.0)	37 (5.7)
**Medicines and CIC, n (%)**	42 (3.9)	31 (7.5) **	24 (15.3) ^††^	7 (2.7)	11 (1.7)
**Number of medicines, n (%)**					
0	870 (81.7)	291 (70.3) ^‡‡^	124 (79.0) ^§§^	167 (65.0)	579 (88.9)
1	161 (15.1)	98 (23.7)	30 (19.1)	68 (26.5)	63 (9.7)
2	27 (2.5)	22 (5.3)	3 (1.9)	19 (7.4)	5 (0.8)
3	7 (0.7)	3 (0.7)	0 (0.0)	3 (1.2)	4 (0.6)
≥4	0 (0.0)	0 (0.0)	0 (0.0)	0 (0.0)	0 (0.0)
Mean [SD]	0.2 [0.5]	0.4 [0.6] **	0.2 [0.5]	0.4 [0.7]	0.1 [0.4]
**Medicine categories, n (%)**					
Anticholinergic drugs only	187 (17.6)	119 (28.7) **	33 (21.0) ^††^	86 (33.5)	68 (10.4)
Beta 3-agonists only	1 (0.1)	1 (0.2)	0 (0.0)	1 (0.4)	0 (0.0)
Other (oral agents) only	3 (0.3)	1 (0.2)	0 (0.0)	1 (0.4)	2 (0.3)
Anticholinergic drugs/beta 3-agonists	4 (0.4)	2 (0.5)	0 (0.0)	2 (0.8)	2 (0.3)
Anticholinergic drugs/other (oral agents)	0 (0.0)	0 (0.0)	0 (0.0)	0 (0.0)	0 (0.0)
Beta 3-agonists/other (oral agents)	0 (0.0)	0 (0.0)	0 (0.0)	0 (0.0)	0 (0.0)
Anticholinergic drugs/beta 3-agonists/Other (oral agents)	0 (0.0)	0 (0.0)	0 (0.0)	0 (0.0)	0 (0.0)

** *p* < 0.01 between the spina bifida and non-spina bifida cohorts by the chi-square test or Fisher’s exact test; ^†^ *p* < 0.05, ^††^ *p* < 0.01 between the open spina bifida and spina bifida occulta cohorts by the chi-square test or Fisher’s exact test; ^‡‡^ *p* < 0.01 between the spina bifida and non-spina bifida cohorts regarding the distribution of the number of medicines in a 2 × 5 contingency table by the Fisher’s exact test; ^§§^ *p* < 0.01 between the open spina bifida and spina bifida occulta cohorts regarding the distribution of the number of medicines in a 2 × 5 contingency table by the Fisher’s exact test. CIC, clean intermittent catheterization; NGB, neurogenic bladder; SD, standard deviation.

**Table 4:** Complications in the 12 months of follow-up.

Incidence, n (%)	NGB Cohort (n = 1065)	Spina Bifida			Non-Spina Bifida Cohort (n = 651)
		Total Cohort (n = 414)	(a) Open (n = 157)	(b) Occulta (n = 257)	
Lower urinary tract infection	193 (18.1)	121 (29.2) **	85 (54.1) ^††^	36 (14.0)	72 (11.1)
Urinary incontinence	85 (8.0)	66 (15.9) **	2 (1.3) ^††^	64 (24.9)	19 (2.9)
Hydronephrosis	60 (5.6)	38 (9.2) **	18 (11.5)	20 (7.8)	22 (3.4)
Obstructive uropathy	35 (3.3)	21 (5.1) *	10 (6.4)	11 (4.3)	14 (2.2)
Upper urinary tract infection	35 (3.3)	16 (3.9)	12 (7.6) ^††^	4 (1.6)	19 (2.9)
Vesicoureteral reflux	33 (3.1)	21 (5.1) **	10 (6.4)	11 (4.3)	12 (1.8)
Frequent urination/frequent urination at night	26 (2.4)	11 (2.7)	1 (0.6)	10 (3.9)	15 (2.3)
Urinary retention	24 (2.3)	6 (1.4)	2 (1.3)	4 (1.6)	18 (2.8)
Renal failure	19 (1.8)	9 (2.2)	4 (2.5)	5 (1.9)	10 (1.5)
Sepsis/septicemia	10 (0.9)	2 (0.5)	2 (1.3)	0 (0.0)	8 (1.2)
Extra-renal urinary overflow	0 (0.0)	0 (0.0)	0 (0.0)	0 (0.0)	0 (0.0)

* *p* < 0.05, ** *p* < 0.01 between the spina bifida and non-spina bifida cohorts by the chi-square test or Fisher’s exact test; ^†^ *p* < 0.05, ^††^ *p* < 0.01 between the open spina bifida and spina bifida occulta cohorts by the chi-square test or Fisher’s exact test. NGB, neurogenic bladder.

## Data Availability

The datasets generated and/or analyzed during the current study are not publicly available because the data was obtained from JMDC Inc. but are available from the corresponding author with the permission of JMDC Inc. on reasonable request.

## Supplementary Materials

The following supporting information can be downloaded at: https://www.mdpi.com/article/10.3390/jcm12093191/s1, Table S1: Neurogenic bladder and overactive bladder diagnosis codes; Table S2: OAB drug codes; Table S3: Neurological disease diagnosis codes; Table S4: Diagnoses in the non-spina bifida cohort (n = 651).
