# Supplementary material for: Diagnosis and Treatment of Japanese Children with Neurogenic Bladder: Analysis of Data from a National Health Insurance Database
Source: J Clin Med. 2023 Apr 28;12(9):3191. doi: 10.3390/jcm12093191 (PMC10179246; doi:10.3390/jcm12093191)
Supplement: Supplementary file 1 [file jcm-12-03191-s001.zip › jcm-2292123-supplementary.pdf]

SUPPLEMENTARY MATERIAL

Supplementary Table S1. Neurogenic bladder and overactive bladder diagnosis

Classification	ICD10 Small Classification	ICD10 Small Classification Name	ICD10 Sub Classification	ICD10 Sub Classification Name	Disease Name
NGB	N31	Neuromuscular dysfunction of bladder, not elsewhere classified	N310	Uninhibited neuropathic bladder, not elsewhere classified	• Uninhibited neurogenic bladder
			N311	Reflex neuropathic bladder, not elsewhere classified	• Reflex neurogenic bladder
			N312	Flaccid neuropathic bladder, not elsewhere classified	• Atonic neurogenic bladder<br>• Autonomous neurogenic bladder<br>• Hypotonic bladder
			N319	Neuromuscular dysfunction of bladder, unspecified	• Bladder and rectal disturbance<br>• Neurogenic bladder<br>• Postoperative bladder dysfunction
OAB	N32	Other disorders of bladder	N328	Other specified disorders of bladder	• Overactive bladder

ICD, International Classification of Diseases; NGB, neurogenic bladder; OAB, overactive bladder

Supplementary Table 2. OAB drug codes

ATC codes	Code Descriptions	General Names	Drug Categories
G04D4	Urinary incontinence products	• Imidafenacin<br>• Oxybutynin hydrochloride<br>• Solifenacin succinate<br>• Tolterodine tartrate<br>• Fesoterodine fumarate<br>• Propiverine hydrochloride	• Anticholinergic drugs
		• Vibegron<br>• Mirabegron	• Beta 3-agonists
G04D4	Urinary incontinence products	Flavoxate hydrochloride	• Other (oral agents)
M03A	Muscle relaxants, peripherally acting	Botulinum toxin type A	• Other (injections)

ATC, Anatomical Therapeutic Chemical Classification; OAB, overactive bladder

Supplementary Table 3. Neurological disease diagnosis codes

Classification	ICD10 Small Classification	ICD10 Small Classification Name	ICD10 Sub Classification	ICD10 Sub Classification Name	Disease Name
Spina bifida	Q05	Spina bifida	Q055	Cervical spina bifida without hydrocephalus	• intracranial dermal sinus
			Q056	Thoracic spina bifida without hydrocephalus	• dorsal spina bifida<br>• thoracolumbar spina bifida
			Q057	Lumbar spina bifida without hydrocephalus	• lumbosacral dermal sinus<br>• lumbosacral spina bifida<br>• rupture of lumbar spinal meningocele
			Q058	Sacral spina bifida without hydrocephalus	• sacral spina bifida
			Q059	Spina bifida, unspecified	• araphia<br>• dermal sinus<br>• hydromeningocele<br>• limbus vertebra<br>• meningocele<br>• myelocele<br>• myelomeningocele<br>• rachischisis<br>• spinal cord lipomeningocele
	Q76	Congenital malformations of spine and bony thorax	Q760	Spina bifida occulta	• spina bifida occulta
Hydrocephalus	Q03	Congenital hydrocephalus	Q030	Malformations of aqueduct of Sylvius	• aqueduct stenosis<br>• congenital aqueduct of Sylvius anomaly

Classification	ICD10 Small Classification	ICD10 Small Classification Name	ICD10 Sub Classification	ICD10 Sub Classification Name	Disease Name
			Q031	Atresia of foramina of Magendie and Luschka	• Dandy-Walker syndrome
			Q038	Other congenital hydrocephalus	• foramen of Monro obstruction
			Q039	Congenital hydrocephalus, unspecified	• X-linked hydrocephalus<br>• congenital hydrocephalus<br>• neonatal hydrocephaly
	Q05	Spina bifida	Q054	Unspecified spina bifida with hydrocephalus	• meningocele with hydrocephalus
Spinal cord injury	T09	Other injuries of spine and trunk, level unspecified	T093	Injury of spinal cord, level unspecified	• spinal cord concussion<br>• spinal cord contusion<br>• spinal cord hematoma<br>• spinal cord incomplete injury<br>• spinal cord injury<br>• spinal epidural hematoma<br>• spinal shock<br>• spinal transverse injury
Cerebral palsy	G80	Cerebral palsy	G800	Spastic quadriplegic cerebral palsy	• congenital spastic paralysis<br>• spastic spinal paralysis
			G801	Spastic diplegic cerebral palsy	• cerebral diplegia<br>• cerebral paraplegia
			G802	Spastic hemiplegic cerebral palsy	• congenital hemiplegia<br>• infantile hemiplegia<br>• pediatric hemiplegia

Classification	ICD10 Small Classification	ICD10 Small Classification Name	ICD10 Sub Classification	ICD10 Sub Classification Name	Disease Name
			G803	Dyskinetic cerebral palsy	• athetoid cerebral palsy<br>• congenital athetosis<br>• congenital chorea<br>• double athetosis<br>• double athetosis<br>• dyskinetic cerebral palsy
			G804	Ataxic cerebral palsy	• ataxic cerebral palsy
			G808	Other cerebral palsy	• atonic cerebral palsy<br>• congenital paraplegia<br>• congenital quadriplegia<br>• mixed cerebral palsy syndrome
			G809	Cerebral palsy, unspecified	• Little disease<br>• cerebral palsy
Meningitis	G03	Meningitis due to other and unspecified causes	G030	Nonpyogenic meningitis	• aseptic meningitis
			G031	Chronic meningitis	• chronic meningitis
			G032	Benign recurrent meningitis [Mollaret]	• Mollaret meningitis
			G039	Meningitis, unspecified	• adhesive arachnoiditis<br>• arachnoiditis<br>• hypertrophic pachymeningitis<br>• leptomeningitis<br>• meningitis<br>• occlusive meningitis<br>• optical neuromeningitis<br>• pachymeningitis<br>• pseudomeningitis<br>• spinal meningitis

Classification	ICD10 Small Classification	ICD10 Small Classification Name	ICD10 Sub Classification	ICD10 Sub Classification Name	Disease Name
Brain tumor	D43	Neoplasm of uncertain or unknown behaviour of brain and central nervous system	D430	Neoplasm of uncertain or unknown behaviour of brain and central nervous system/Brain, supratentorial	• diencephalic tumor<br>• frontal lobe tumor<br>• hypothalamic neoplasm<br>• occipital lobe tumor<br>• parasellar tumor<br>• parietal lobe tumor<br>• supratentorial brain tumor<br>• temporal lobe tumor<br>• thalamic tumor<br>• third ventricle tumor
			D431	Neoplasm of uncertain or unknown behaviour of brain and central nervous system/Brain, infratentorial	• brainstem tumor<br>• bulbar hemangioblastoma<br>• bulbar tumor<br>• cerebellar hemangioblastoma<br>• cerebellar neoplasm<br>• cerebellar vermis tumor<br>• cerebellopontine angle tumor<br>• foramen magnum tumor<br>• fourth ventricle tumor<br>• infratentorial brain tumor<br>• posterior fossa tumor

Classification	ICD10 Small Classification	ICD10 Small Classification Name	ICD10 Sub Classification	ICD10 Sub Classification Name	Disease Name
			D432	Neoplasm of uncertain or unknown behaviour of brain and central nervous system/Brain, unspecified	• brain tumor<br>• congenital brain tumor<br>• cranial nerve neoplasm<br>• dysembryoplastic neuroepithelial tumor<br>• intracranial teratoma<br>• intraventricular tumor<br>• multiple brain tumor<br>• myxopapillary ependymoma<br>• papillary ependymoma<br>• subependymal giant cell astrocytoma<br>• subependymoma
			D433	Neoplasm of uncertain or unknown behaviour of brain and central nervous system/Cranial nerves	• acoustic neuroma<br>• chiasmal tumor<br>• olfactory tumor<br>• optic disc tumor<br>• optic nerve neoplasm

Classification	ICD10 Small Classification	ICD10 Small Classification Name	ICD10 Sub Classification	ICD10 Sub Classification Name	Disease Name
			D434	Neoplasm of uncertain or unknown behaviour of brain and central nervous system/Spinal cord	• intramedullary spinal cord tumor<br>• cauda equina tumor<br>• cervical cord tumor<br>• dumbbell shaped tumor<br>• extradural spinal cord tumor<br>• intraspinal teratoma<br>• intraspinal tumor<br>• spinal cord tumor<br>• spinal hemangioblastoma<br>• spinal intradural-extramedullary angioblastoma<br>• thoracic cord tumor
Myelitis	G04	Encephalitis, myelitis and encephalomyelitis	G040	Acute disseminated encephalitis	• acute disseminated encephalomyelitis<br>• postvaccinal encephalomyelitis<br>• vaccination encephalitis
			G042	Bacterial meningoencephalitis and meningomyelitis, not elsewhere classified	• purulent cerebral meningitis<br>• purulent myelitis
			G048	Other encephalitis, myelitis and encephalomyelitis	• Rasmussen's encephalitis<br>• acute cerebellar ataxia<br>• anti-NMDA receptor encephalitis<br>• atopic myelitis<br>• autoimmune limbic encephalitis<br>• postinfectious encephalitis<br>• postinfectious encephalomyelitis

Classification	ICD10 Small Classification	ICD10 Small Classification Name	ICD10 Sub Classification	ICD10 Sub Classification Name	Disease Name
			G049	Encephalitis, myelitis and encephalomyelitis, unspecified	• acute ascending myelitis<br>• acute myelitis<br>• chronic encephalitis<br>• chronic myelitis<br>• compression myelitis<br>• encephalitis<br>• encephalomyelitis<br>• limbic encephalitis<br>• meningoencephalitis<br>• meningomyelitis<br>• myelitis<br>• myelomeningitis<br>• polymyeloradiculitis<br>• sclerosing myelitis<br>• secondary encephalitis<br>• ventriculitis
	G37	Other demyelinating diseases of central nervous system	G373	Acute transverse myelitis in demyelinating disease of central nervous system	• acute transverse myelitis
					• transverse myelitis

ICD, International Classification of Diseases

Supplementary Table 4. Diagnoses in the non-spina bifida cohort (n = 651)

Diagnosis	n	%
Cerebral palsy	475	73.0
Brain tumor	50	7.7
Meningitis	47	7.2
Myelitis	22	3.4
Hydrocephalus/cerebral palsy	16	2.5
Hydrocephalus	10	1.5
Cerebral palsy/brain tumor	8	1.2
Cerebral palsy/myelitis	6	0.9
Spinal cord injury	4	0.6
Cerebral palsy/meningitis/myelitis	3	0.5
Spinal cord injury/brain tumor	2	0.3
Meningitis/myelitis	2	0.3
Brain tumor/myelitis	2	0.3
Hydrocephalus/cerebral palsy/meningitis	1	0.2
Hydrocephalus/cerebral palsy/brain tumor	1	0.2
Spinal cord injury/cerebral palsy	1	0.2
Spinal cord injury/meningitis	1	0.2
